# A Review of Agentic AI in Cybersecurity: Cognitive Autonomy, Ethical Governance, and Quantum-Resilient Defense

**DOI:** 10.12688/f1000research.169337.1

**Published:** 2025-09-01

**Authors:** IBRAHIM ADABARA, Bashir Olaniyi Sadiq, Aliyu Nuhu Shuaibu, Yale Ibarahim Danjuma, Maninti Venkateswarlu

**Affiliations:** 1 Information Technology, Kampala International University - Western Campus, Bushenyi, Western Region, Uganda; 2 of Electrical Telecommunication and Computer Engineering, Kampala International University - Western Campus, Bushenyi, Western Region, Uganda

**Keywords:** Agentic Artificial Intelligence, Cybersecurity, Cognitive Autonomy, Ethical Governance, Quantum-Resilient Systems, AI Threat Mitigation, Autonomous Cyber Defense

## Abstract

Agentic Artificial Intelligence (AAI) refers to autonomous, adaptable, and goal-directed systems capable of proactive decision-making in dynamic environments. These agentic systems extend beyond reactive AI by leveraging cognitive architectures and reinforcement learning to enhance adaptability, resilience, and self-sufficiency in cybersecurity contexts. As cyber threats grow in sophistication and unpredictability, Agentic AI is rapidly becoming a foundational technology for intelligent cyber defense, enabling capabilities such as real-time anomaly detection, predictive threat response, and quantum-resilient protocols. This narrative review synthesizes literature from 2005 to 2025, integrating academic, industry, and policy sources across three thematic pillars: cognitive autonomy, ethical governance, and quantum-resilient defense. The review identifies key advancements in neuromorphic architectures, cross-jurisdictional governance models, and hybrid defense systems that adapt to evolving threat landscapes. It also exposes critical challenges, including dual-use risks, governance interoperability, and preparedness for post-quantum security. This work contributes a multi-dimensional conceptual framework linking governance mechanisms to operational practice, maps resilience strategies across conventional and quantum vectors, and outlines a forward-looking roadmap for secure, ethical, and adaptive deployment of Agentic AI in cybersecurity. The synthesis aims to support policymakers, developers, and security practitioners in navigating the accelerating convergence of autonomy, security, and AI ethics.

## 1. Introduction

### 1.1 Background

Agentic Artificial Intelligence refers to autonomous systems capable of pursuing complex, goal-directed tasks with minimal human intervention, distinguished by adaptability, decision-making autonomy, and operational resilience in dynamic environments. ^1^ Unlike traditional AI models that operate reactively, agentic AI proactively plans, adapts, and executes workflows, aligning with theories of autonomy and adaptivity central to intelligent agent design. ^2^ In the context of cybersecurity, this capability is increasingly valuable due to the sophistication, speed, and unpredictability of emerging threats. The scarcity of skilled cybersecurity professionals, combined with the growing complexity of attack surfaces, creates a pressing need for autonomous cyber-defense agents capable of sensing, reasoning, acting, and learning without constant oversight. ^3^ These agents leverage autonomy to maintain operational continuity, even when communication with human operators is degraded or adversarial manipulation attempts are underway. ^4^ The principles underlying agentic AI in cyber defense intersect with resilience engineering and sociotechnical systems theory, emphasizing adaptability, fault tolerance, and human-machine collaboration in uncertain conditions. ^5^ From a sociotechnical perspective, designing AI that can augment human decision-making while preserving ethical governance aligns with broader goals of ensuring trust, accountability, and interoperability across systems. ^6^

### 1.2 Problem context

The cybersecurity threat landscape has undergone rapid transformation, driven by increasingly sophisticated attack vectors such as advanced persistent threats (APTs), polymorphic malware, and adversarial machine learning (AML) attacks. Conventional defense models, often reliant on static rules, perimeter-based protections, and human-centric incident response, are proving inadequate in addressing these evolving risks. ^7^ These legacy systems struggle with detection latency, limited adaptability, and the inability to effectively counter stealthy, long-term intrusions such as APTs. ^8^ Emerging cyber threats increasingly exploit gaps in static defense mechanisms, bypassing traditional intrusion detection and prevention systems through zero-day vulnerabilities and social engineering tactics. ^9^ Furthermore, the expanding digital attack surface amplified by cloud adoption, IoT proliferation, and distributed architectures has introduced complexity beyond the operational scope of conventional security strategies. ^10^ To address these limitations, the cybersecurity domain is increasingly exploring autonomous, adaptive defense paradigms that integrate AI for real-time threat detection, proactive risk mitigation, and resilience-building. ^11^ This paradigm shift highlights the urgent need for more dynamic and intelligent frameworks capable of evolving alongside the threats they are designed to counter.

### 1.3 Role of agentic AI

AAI introduces a paradigm shift in cybersecurity by enabling systems to operate with cognitive autonomy, adaptability, and proactive decision-making. Unlike traditional AI, which responds to predefined prompts or static rules, Agentic AI autonomously pursues complex goals, learns from dynamic environments, and adapts strategies in real-time. ^1^ This autonomy allows for advanced threat detection and response capabilities, especially in contexts where rapid, unsupervised decision-making is critical to system survival. In cybersecurity, the role of Agentic AI extends beyond detection to include autonomous mitigation, strategic defense orchestration, and quantum-resilient risk anticipation. Cognitive architectures integrated with reinforcement learning and neuromorphic frameworks enable these agents to emulate human-like reasoning while maintaining superior scalability and speed. ^12^

By leveraging quantum-enhanced learning, Agentic AI systems can improve decision accuracy and significantly reduce training time, thereby accelerating adaptation to novel threats. Agentic AI also introduces embedded governance potential, where ethical oversight mechanisms can be directly integrated into autonomous workflows to ensure compliance with fairness, transparency, and accountability principles. ^13^ Furthermore, autonomous cyber-defense agents (AICAs) demonstrate how distributed, goal-driven AI entities can defend compromised or isolated networks without human intervention, closing critical response gaps caused by communication delays or resource shortages. ^3^ By combining adaptability, proactive resilience, and embedded ethical governance, Agentic AI offers a robust framework for evolving beyond reactive cybersecurity models toward self-sustaining, anticipatory defense ecosystems.

### 1.4 Emerging risks

1.4.1 Technical risk vectors

The rapid integration of Agentic AI into cybersecurity introduces a complex range of technical vulnerabilities that exceed those addressed by traditional defense models. One of the most critical areas is adversarial AI, where attackers exploit weaknesses in learning models through data poisoning, evasion tactics, and generative deepfakes to mislead or disable autonomous agents. ^14^ These techniques compromise both the integrity and trustworthiness of agentic systems, especially those relying on real-time inference or adaptive learning. Moreover, as agentic architectures become more complex and data-driven, model inversion and extraction attacks represent serious threats to proprietary model assets and user privacy. The deployment of online learning or few-shot adaptation further increases exposure to manipulation, making it easier for adversaries to steer system behavior toward compromised or suboptimal states over time. ^15^

**Figure 1.**
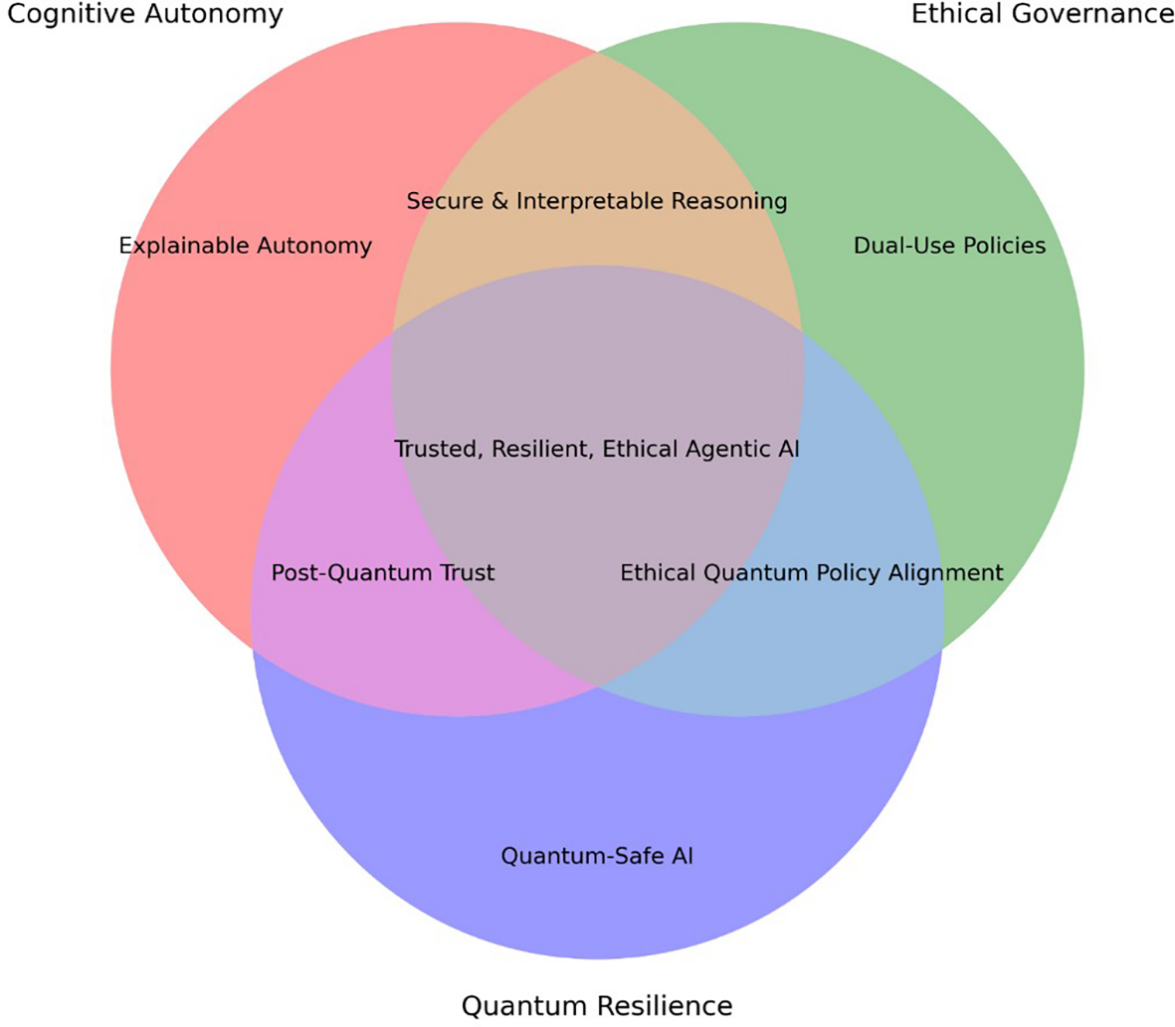
Conceptual map: Intersections among cognitive autonomy, ethical governance, and quantum resilience.

Compounding these risks is the advent of quantum computing, which presents an existential threat to the cryptographic underpinnings of secure communication systems. Algorithms like Shor’s make traditional encryption methods such as RSA and ECC potentially obsolete, raising the real possibility of “harvest now, decrypt later” scenarios. ^16^ In this environment, autonomous AI agents handling secure credentials or key management functions could become high-value targets, necessitating quantum-resilient architectures and post-quantum cryptographic protocols. Together, these vectors demonstrate the urgent need for resilience-by-design practices in agentic systems. This includes adversarial robustness, model verifiability, runtime threat detection, and secure update mechanisms. The conceptual relationships among cognitive autonomy, governance requirements, and quantum resilience are visualized in Figure 1, illustrates the conceptual intersections among the three foundational pillars of agentic AI in cybersecurity: Cognitive Autonomy, Ethical Governance, and Quantum Resilience. Each pillar contains distinct thematic elements, while the overlap zones represent shared challenges and synergistic opportunities such as trust calibration, secure autonomy, and dual-use governance strategies.

1.4.2 Governance and dual-use challenges

Beyond technical complexity, Agentic AI introduces major challenges in governance, ethical oversight, and strategic misuse, particularly in the context of dual-use applications. These systems, by their autonomy and learning capability, blur the boundary between defensive protection and offensive exploitation. The dual-use dilemma is a central concern: tools originally designed for cyber defense, such as autonomous intrusion detection or self-healing systems, can be repurposed for offensive purposes, such as autonomous probing, system infiltration, or self-replicating malware agents. ^17^ The accessibility of these capabilities, combined with the lack of attribution in cyber warfare, creates scenarios where unintended escalation or covert cyber operations may proliferate. At the same time, current governance frameworks often lack the transparency, accountability, and international harmonization required to manage such systems effectively. Many agentic systems operate as “black boxes” with limited explainability, complicating compliance with ethical principles like traceability, justice, and human oversight as outlined in regulatory frameworks like the EU AI Act or ISO/IEC AI standards. ^18^ The regulatory lag, the time gap between technological advancement and legal or ethical controls, further increases governance risks. In this void, actors can deploy powerful agentic AI without sufficient safeguards, especially across jurisdictions with uneven oversight capacity. The risk is magnified in contexts with geopolitical asymmetry, where state and non-state actors exploit legal grey zones to develop and deploy ethically ambiguous capabilities.

**Figure 2.**
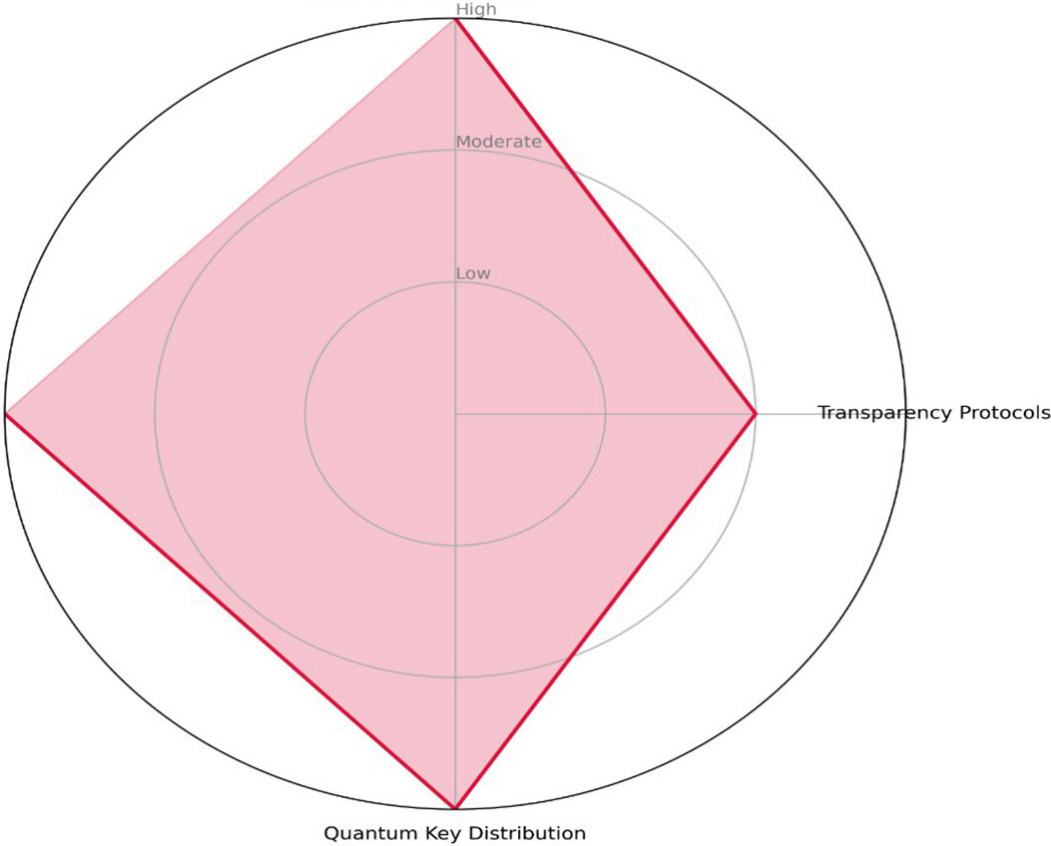
Dual-use risk overlay diagram-visual overlay showing where misuse potential exists across cognitive autonomy, ethical governance, and quantum resilience.

As the autonomy and sophistication of agentic systems grow, governance must evolve beyond static compliance to embrace dynamic, lifecycle-aware models of control. These should include ethical risk forecasting, operational transparency, and shared international norms. These governance and ethical challenges further reinforce the need for agentic AI frameworks that are not only secure and adaptable, but also normatively grounded. The breadth and severity of dual-use and governance risks are illustrated in Figure 2, highlights the dual-use risk zones across the three thematic pillars of agentic AI in cybersecurity. It maps areas where technologies designed for defense or oversight can be repurposed maliciously such as explainability for deception, autonomy for offensive decision-making, or quantum tools for surveillance evasion. This overlay reinforces the ethical imperative of governance-aware system design.

### 1.5 Knowledge gaps

Despite rapid advancements in agentic AI, significant knowledge gaps remain in governance, integration, and risk management. Research indicates that AI governance (AIG) is still in an emergent state, with a limited understanding of how to effectively implement ethical principles into operational practices. Critical deficiencies include a lack of contextual awareness, uncertainty regarding the effectiveness of regulations, and insufficient operationalization of governance processes. ^19^ The trustworthiness of AI systems is hindered by the absence of robust metrics for human-centric risks, such as bias, misinformation, and privacy erosion. Existing frameworks often focus on technical vulnerabilities while neglecting socio-psychological threats and interdisciplinary collaboration needs. ^20^ From a security perspective, knowledge gaps persist in addressing quantum-era threats and building adaptive, sector-specific cybersecurity strategies. Emerging studies recommend integrating quantum-ready encryption and adaptive risk models, but practical implementation remains limited. ^21^ Furthermore, there is an identified AI knowledge gap at the academic and industry level, where the pace of system development outstrips the number of empirical studies characterizing AI behavior. Bridging this requires cross-disciplinary collaboration and institutional incentives to promote systematic evaluation of AI systems. ^22^ In addition, sector-specific readiness varies widely. Furthermore, in healthcare, clinicians often lack adequate AI literacy, limiting their ability to evaluate, deploy, and monitor AI tools effectively. Structured educational frameworks and regulatory alignment are needed to ensure safe and ethical adoption. ^23^ Similarly, global and regional disparities in AI policy readiness hinder cohesive governance strategies, particularly in resource-limited regions. ^24^

Overall, addressing these knowledge gaps will require:

• Operationalizing AI governance frameworks with measurable outcomes.
• Developing socio-technical trustworthiness metrics.
• Preparing for quantum-era cybersecurity challenges.
• Expanding empirical research on AI behavior.
• Building sector-specific education and readiness programs.

### 1.6 Rationale for review type

The rapidly evolving nature of agentic AI in cybersecurity necessitates a synthesis approach that captures diverse perspectives, emerging trends, and multidisciplinary insights. A narrative review is particularly suited for this purpose, as it allows the integration of findings from heterogeneous sources, including both peer-reviewed and grey literature, thereby providing a richer and more contextual understanding than purely systematic approaches. Narrative reviews are valuable for capturing thematic breadth, exploring conceptual linkages, and accommodating evolving terminologies and methodologies that may not yet be standardized in empirical databases. ^25^ Additionally, narrative review methodologies are effective in mapping emerging domains where empirical evidence may be limited, fragmented, or in non-traditional formats. This flexibility is essential in fields like AI-enhanced cybersecurity, where insights from technical reports, policy papers, and case studies can be as informative as academic journal articles. ^26^ The narrative review format also enables the identification of thematic gaps and conceptual trends that can guide future research and policy development. ^27^ Moreover, in multidisciplinary contexts, such as the convergence of AI, cybersecurity, governance, and ethics, a narrative review can synthesize perspectives across domains without being constrained by rigid inclusion criteria that might exclude innovative or early-stage work. ^28^ This approach ensures that the review remains responsive to the dynamic and rapidly changing landscape of cyber threats and AI capabilities, supporting a holistic and forward-looking synthesis.

### 1.7 Review objective & scope

The objective of this review is to synthesize and critically analyze the evolution, governance, and resilience dimensions of AAI within cybersecurity, emphasizing cognitive autonomy, ethical governance, and quantum-resilient defense strategies. The review aims to bridge conceptual frameworks with practical applications, drawing from interdisciplinary insights across computer science, security studies, ethics, and emerging quantum technologies.

This study is guided by four central research questions:

**RQ1:** What are the prevailing design patterns and architectural principles in agentic AI for cybersecurity?

**RQ2:** How are ethical governance mechanisms implemented to align agentic AI with regulatory and compliance frameworks?

**RQ3:** What strategies are being developed for resilience against both conventional and quantum-era threats in agentic AI systems?

**RQ4:** What are the primary implementation barriers and enabling factors for deploying agentic AI in cybersecurity contexts?

These questions are anchored in three thematic pillars (1) Cognitive Autonomy, (2) Ethical Governance, and (3) Quantum-Resilient Defense, which provide a structured lens for thematic synthesis. Prior research has highlighted the necessity of integrating autonomy with robust cognitive architectures, ^29^ addressing security vulnerabilities in autonomous agents, ^4^ and developing resilience frameworks that anticipate quantum-era disruptions. ^30^ The review’s scope spans literature from 2005 to 2025, integrating academic, industry, and policy perspectives. This time frame captures the formative years of agentic AI conceptualization, recent governance reforms, and the emergence of quantum-resilient strategies. By adopting a narrative review approach, the study allows for thematic breadth, inclusion of grey literature, and cross-domain insights essential for understanding the socio-technical implications of deploying agentic AI in complex cybersecurity ecosystems.

### 1.8 Terminology & scope clarifications

Agentic AI refers to artificial intelligence systems capable of autonomous decision-making, adaptability, and goal-directed reasoning, often integrating cognitive frameworks and reinforcement learning to operate with minimal human intervention in dynamic environments. ^12^ These systems extend beyond traditional automation by incorporating higher-order cognitive functions such as self-reflection, context awareness, and adaptive problem-solving.

Cognitive Autonomy denotes the capacity of an AI agent to independently process information, learn from diverse experiences, and generate novel solutions, often drawing on quantum-inspired or neuromorphic architectures to address uncertainty and incomplete knowledge. ^31^ This form of autonomy emphasizes not only decision accuracy but also the system’s ability to navigate ethical dilemmas and conflicting objectives. ^32^

Ethical Governance in Agentic AI involves the establishment of oversight frameworks, policies, and technical safeguards that ensure transparency, accountability, and alignment with human values. Models such as decentralized governance systems have been proposed to address the challenges of regulating highly autonomous AI agents, leveraging tools like blockchain, smart contracts, and verifiable identity protocols. ^33^

Quantum-Resilient Defense refers to cybersecurity strategies designed to withstand quantum-era threats, incorporating post-quantum cryptographic algorithms and AI-based resilience mechanisms. By embedding quantum-resistant features into autonomous agents, systems can maintain security integrity even in the face of future quantum computing capabilities. ^34^

For this review, the scope is limited to AI systems with demonstrable autonomy and decision-making capacity that directly influence cybersecurity operations. Out of scope are purely algorithmic tools lacking adaptive or cognitive features, as well as general discussions of AI ethics unrelated to security contexts. The thematic pillars guiding this review, Cognitive Autonomy, Ethical Governance, and Quantum-Resilient Defense, serve as the structural framework for synthesizing literature across academic, industrial, and policy domains.

## 2. Methodology (Narrative review approach)

### 2.1 Scope

This review adopts a narrative review methodology to provide a broad yet thematically focused synthesis of literature on Agentic AI in cybersecurity. Unlike systematic reviews that rely on strict inclusion and exclusion criteria, narrative reviews enable the integration of diverse evidence sources, including peer-reviewed research, grey literature, and conceptual papers, to capture the full spectrum of emerging developments and perspectives. ^35^ This approach is particularly valuable in rapidly evolving fields such as AI-driven cybersecurity, where technological innovations, threat landscapes, and governance frameworks shift quickly.

• The scope of this review encompasses technical, governance, and socio-ethical dimensions of Agentic AI applications in cyber defense. Specifically, it covers.
• Technological foundations, including autonomy, adaptability, and cognitive reasoning architectures for cybersecurity defense agents. ^36^
• Operational applications such as automated threat detection, incident response, and quantum-resilient defense strategies.
• Governance and ethical considerations addressing transparency, bias mitigation, and accountability in autonomous systems. ^37^
• Cross-domain integration examining lessons from other sectors such as healthcare and finance, where AI adoption has generated both efficiency gains and equity concerns. ^38^

By framing the scope across technological, operational, and governance dimensions, this review aims to deliver a multidimensional perspective that is relevant to both academic inquiry and practical cybersecurity policy-making.

### 2.2 Search strategy

The search strategy for this narrative review was designed to ensure breadth, depth, and methodological rigor, while maintaining transparency in decision-making. Following recommendations for narrative review structuring, the process began by identifying core concepts: Agentic AI, cybersecurity, and emerging governance frameworks, and then expanding the search using Boolean operators and wildcard symbols to capture relevant variations in terminology. ^39^ Key academic databases, including IEEE Xplore, Scopus, Web of Science, and ACM Digital Library, were targeted to capture peer-reviewed literature, complemented by grey literature searches in organizational reports, policy briefs, and conference proceedings. Boolean combinations were crafted to merge technical (for instance, “machine learning,” “autonomous agents,” “threat detection”) and contextual terms (for instance, “cyber policy,” “governance frameworks,” “critical infrastructure”), a practice shown to enhance comprehensiveness in cybersecurity reviews. ^40^ While the complete Boolean search strings and refinement process are available in the supplementary dataset. ^41^

The search process was iterative rather than linear, with periodic refinement of terms as familiarity with the literature increased, consistent with best practices for narrative synthesis. ^42^ In line with recent methodological advancements, generative AI tools were selectively used to identify thematic clusters and suggest additional terms, increasing the efficiency of retrieval without replacing researcher judgment. ^43^ To ensure relevance, retrieved documents were screened against predefined thematic pillars and research questions, and reference chaining was employed to locate key works cited by foundational studies. This combination of database querying, grey literature capture, and citation chasing is recognized as a robust approach for mapping complex, interdisciplinary domains like AI-enabled cybersecurity. ^44^

### 2.3 Coverage period & source types

This review encompasses literature published from the early 2010s through 2025 to capture the rapid evolution of agentic AI, cybersecurity resilience, and quantum-readiness paradigms. The timeframe aligns with the emergence of AI-enabled cyber threat models, governance frameworks, and ethical discourses that have shifted significantly in the last decade. ^45^ Source types include peer-reviewed journal articles, conference proceedings, authoritative narrative reviews, systematic literature reviews, and selected grey literature from reputable institutional or policy sources to ensure both academic rigor and practical applicability. ^46^ By incorporating both traditional academic studies and non-traditional but credible reports, the review benefits from a broader evidence base that reflects ongoing developments in highly dynamic technological landscapes. ^37^ Given the complexity and cross-domain nature of agentic AI applications, the inclusion of diverse source types enables the integration of conceptual, empirical, and practice-oriented perspectives. ^47^ This approach ensures that the review remains comprehensive while reflecting the multidisciplinary realities of AI-driven cybersecurity systems.

### 2.4 Eligibility criteria

To ensure methodological rigor and relevance, this review adopted explicit inclusion and exclusion criteria grounded in established guidance for narrative and scoping reviews. ^48^

Inclusion criteria comprised:

• Peer-reviewed journal articles, authoritative narrative reviews, and high-quality grey literature published between 2010 and 2025.
• Studies addressing agentic AI in cybersecurity, quantum readiness, governance, or ethical frameworks.
• Empirical, conceptual, or policy-oriented works providing actionable insights for cross-domain resilience.

Exclusion criteria included:

• Studies not in English.
• Non-scholarly content lacking methodological transparency or relevance to the thematic scope.
• Redundant studies where newer or more comprehensive sources covered the same ground.

The adoption of clearly defined eligibility parameters improves transparency, reproducibility, and credibility, while reducing bias in literature selection. ^49^ Such rigor ensures that the review accurately reflects both the breadth and depth of the evidence base on emerging AI-driven cybersecurity challenges. ^50^

### 2.5 Review process flow

**Figure 3.**
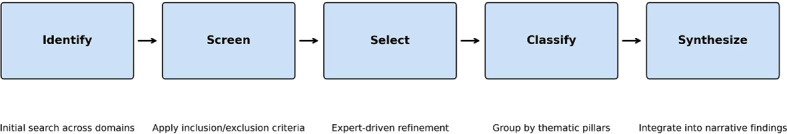
Narrative review process flow.

The review adopted a five-stage process to ensure a structured yet adaptable narrative synthesis. Identify: Relevant literature was located through systematic database searches (IEEE Xplore, ACM Digital Library, Scopus, Web of Science) and targeted grey literature repositories. Boolean keyword clustering was employed to maximize thematic coverage. ^26^ Screen: Initial screening involved title and abstract review to remove irrelevant materials, with emphasis on aligning studies to the thematic pillars: cognitive autonomy, ethical governance, and quantum-resilient defense. ^51^ Select: Full-text reviews were conducted for eligible papers, guided by predefined inclusion/exclusion criteria to maintain consistency and methodological transparency. ^48^ Classify: Selected works were coded according to their thematic relevance (RQ1-RQ4), type (conceptual, empirical, policy), and domain focus, enabling cross-pillar linkages and identification of evidence clusters. ^52^ Synthesize: Findings were integrated into a narrative format that preserved contextual richness while drawing comparative insights across studies, thereby supporting conceptual model development and identification of knowledge gaps. ^43^ This sequential flow allowed the review to maintain methodological rigor while remaining responsive to emergent concepts and evolving research directions in agentic AI cybersecurity. Figure 3. Process flow of the narrative review methodology. This includes five sequential stages: Identify, Screen, Select, Classify, and Synthesize. The diagram emphasizes thematic sorting, expert validation, and cross-domain synthesis unique to this review’s integrative approach.

### 2.6 Thematic synthesis

The thematic synthesis process organized findings across the four research questions (RQ1-RQ4), allowing for a structured integration of cognitive autonomy, ethical governance, and quantum-resilient defense perspectives. This approach drew on principles of thematic analysis while incorporating AI-assisted literature mapping to identify patterns and conceptual linkages within and across thematic pillars. ^52^ For RQ1 (design patterns in Agentic AI cybersecurity), extracted data were coded into recurring categories such as hybrid human -agent architectures, neuromorphic cognitive frameworks, and reinforcement learning variants, with emphasis on domain-specific applications. ^12^ For RQ2 (ethical governance mechanisms), synthesis mapped governance approaches from global frameworks (e.g., NIST AI RMF, ISO/IEC standards) against observed implementation practices, revealing alignment gaps and variations in accountability structures. ^21^ For RQ3 (threat resilience strategies), themes encompassed both cryptographic and non-cryptographic methods, including quantum-resistant protocols, AI model integrity safeguards, and resilience-by-design engineering principles. ^53^ For RQ4 (implementation barriers and enablers), synthesized evidence highlighted the influence of cost constraints, skills shortages, and policy fragmentation as major inhibitors, while collaborative ecosystems and sector-specific funding emerged as enabling factors. ^54^ Cross-pillar synthesis revealed strong interdependencies: advancements in cognitive autonomy often necessitated governance innovation, and quantum-resilient security strategies were most effective when embedded within ethically-aligned agentic frameworks.

### 2.7 Methodological limitations

While the review’s methodology aimed for comprehensiveness, several inherent limitations must be acknowledged. First, AI-focused cybersecurity literature often exhibits rapid obsolescence due to evolving threat landscapes and technological advancements, meaning findings may lose relevance quickly. ^55^ Second, AI-based threat detection studies frequently suffer from data bias and limited generalizability, as training datasets may not fully represent emerging or rare attack vectors, impacting external validity. ^56^ Third, reliance on simulation and controlled environments in many evaluations constrains ecological validity, as real-world deployment introduces unpredictable system, human, and adversarial factors. ^57^ Fourth, adversarial vulnerabilities in AI models remain underreported in empirical literature, creating a blind spot in systematic reviews of security performance. ^58^ Finally, language and indexing bias may have excluded relevant non-English studies or grey literature, potentially narrowing the thematic breadth of the synthesis. ^59^ These limitations highlight the need for ongoing updates, diversified data sources, and inclusion of real-world case validations to strengthen future reviews.

## 3. State of the art

### 3.1 Evolution of agentic AI in cybersecurity

The progression of Agentic Artificial Intelligence (AI) in cybersecurity reflects a broader shift in AI from reactive, rule-based systems toward autonomous, goal-driven agents capable of adaptive decision-making. Early AI systems in cybersecurity focused on static pattern matching and predefined rule sets, which were effective for known threats but struggled against novel attack vectors. The rise of Generative AI marked a turning point by enabling large-scale language and vision models that could reason and adapt dynamically, laying the groundwork for more autonomous, agentic capabilities. ^60^ The adoption of Agentic AI has been propelled by its ability to integrate cognitive autonomy, persistent memory, and multi-agent collaboration features that allow for continuous monitoring, adaptive learning, and proactive threat mitigation in complex digital environments. ^61^ This evolution has also been shaped by the incorporation of cognitive skills modules tailored to specific domains, improving decision-making precision and scalability. ^62^ In cybersecurity specifically, Agentic AI has transformed defensive strategies by reimagining frameworks like the cyber kill chain, integrating real-time threat intelligence, and embedding ethical governance into automated responses. ^63^ These advances address the limitations of conventional defense by enabling autonomous orchestration of countermeasures against Advanced Persistent Threats (APTs) and other sophisticated attack forms. ^64^

The historical trajectory of Agentic AI in cybersecurity can be viewed in distinct phases:

• Rule-Based Automation (Pre-2010s): Reliance on static rules and expert systems.
• Machine Learning Integration (2010-2020): Incorporation of supervised and unsupervised learning for anomaly detection.
• Generative & Hybrid AI (2020-2023): Rise of LLMs and multi-modal AI as adaptive reasoning engines.
• Agentic AI Era (2023 - Present): Fully autonomous, collaborative agents with embedded governance and quantum-resilient readiness. ^65^

This evolution has redefined the cybersecurity domain by shifting the paradigm from reactive defense to proactive, ethically governed autonomy capable of anticipating and countering emerging threats before they escalate.

### 3.2 Current landscape

The current landscape of Agentic AI in cybersecurity reflects rapid technological convergence, diverse sectoral applications, and growing industry adoption. Key technologies underpinning this domain include autonomous decision-making architectures, multi-agent coordination systems, and AI-enhanced threat intelligence platforms. Recent developments demonstrate how Agentic AI is increasingly integrated with complementary technologies such as blockchain to enhance trust, transparency, and resilience in cyber defense systems, leading to significant improvements in anomaly detection accuracy and incident response efficiency. ^66^ Industry uptake spans multiple sectors. Financial institutions leverage Agentic AI for fraud detection and high-frequency threat analysis, while healthcare organizations use it to protect sensitive patient data and enforce fine-grained access control policies. ^66^ Governmental agencies have also begun integrating agentic and frontier AI capabilities into critical infrastructure protection, particularly in enhancing real-time monitoring and automated incident response. ^63^ The technology ecosystem is evolving toward proactive and perpetual learning systems capable of adapting to new threat vectors without human intervention. This shift is accompanied by an increase in deployment scale, ranging from organizational-level solutions to national cyber defense initiatives. ^67^ However, while adoption rates are accelerating, challenges persist, particularly regarding ethical governance, interoperability across sectors, and the quantum readiness of deployed systems. To provide an overview of the academic and grey literature informing this review, Table 1 summarizes key studies across relevant domains. Each entry includes author(s), year, national/regional context, domain of application, and the study’s core contribution to the agentic AI and cybersecurity landscape. This table offers a snapshot of the diversity and interdisciplinary nature of current research, forming the empirical backbone for the thematic synthesis presented in later sections.

**Table 1.** Summary of reviewed studies (authors, year, country, domain, contribution).

Author(s)	Year	Country	Domain	Key contribution
Ratnawita	2025	USA	Adversarial AI	Demonstrated data poisoning threats in autonomous systems
Chimamiwa	2024	South Africa	Autonomous Learning	Analyzed few-shot learning risks in cybersecurity AI
Elmisery et al.	2025	Qatar	Quantum Security	Forecasted quantum threats to agentic AI systems
Dubey et al.	2025	India	Dual-Use Ethics	Explored dual-use risks of defensive agentic AI
Lekota	2024	Nigeria	Governance & Regulation	Mapped oversight gaps in agentic AI governance

### 3.3 Publication trends

**Figure 4.**
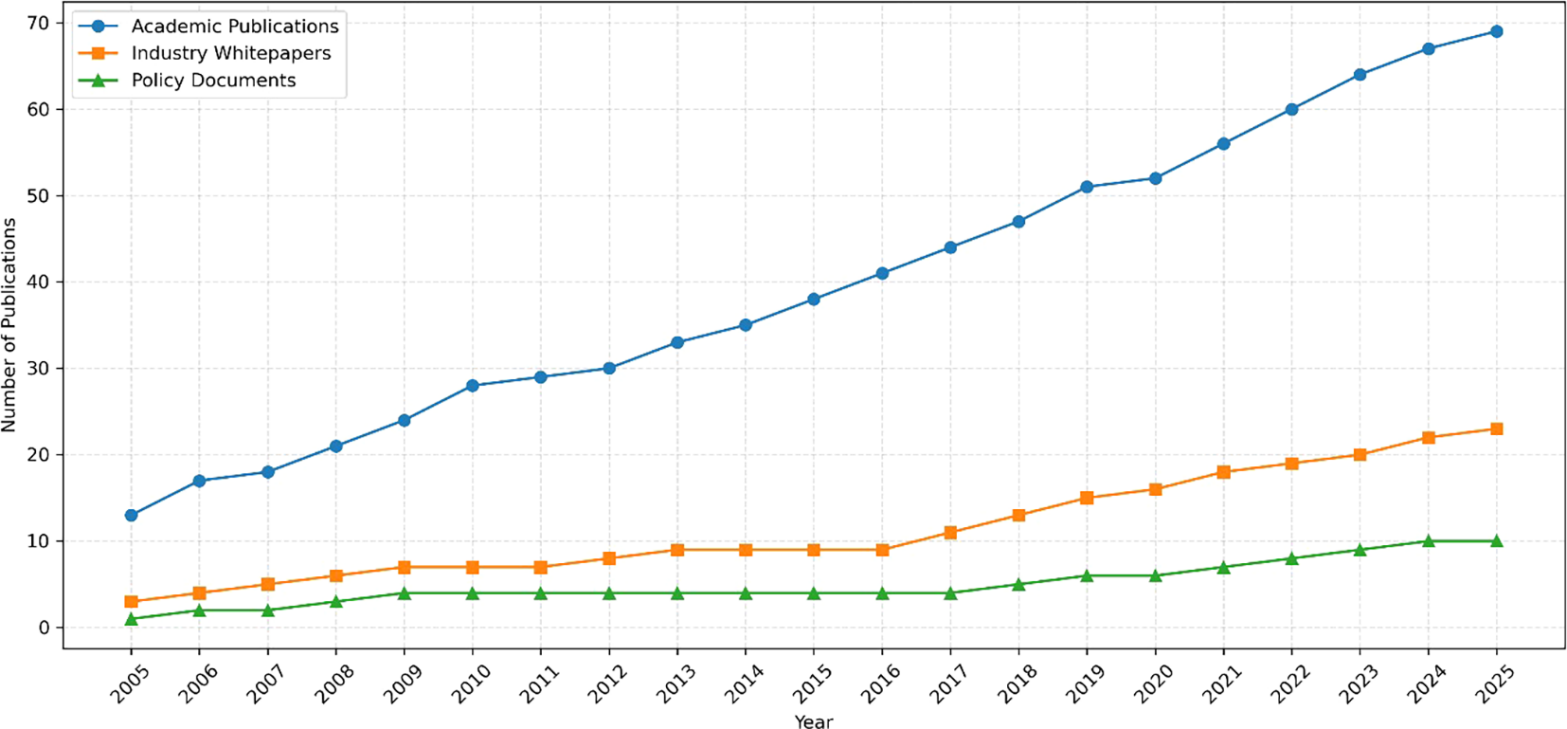
Annual publication trends.

Bibliometric analyses reveal a sustained upward trajectory in scholarly output on agent-based and agentic AI applications in cybersecurity over the past decade. For instance, global publications on agent-based cybersecurity systems have steadily risen since 2013, peaking in 2023 with more than 1,200 articles and over 30,000 citations, reflecting both growing research interest and the field’s expanding influence. ^68^ Similarly, research at the broader AI -cybersecurity nexus has shown remarkable acceleration since 2015, driven by the emergence of machine learning -based intrusion detection, adversarial attack studies, and IoT security solutions. ^69^ Trends have been fueled by factors such as increasing cyber threat sophistication, the proliferation of connected devices, and strategic investment in AI-enhanced security frameworks. The surge also aligns with thematic shifts toward integrating blockchain, quantum computing, and explainable AI in security architectures, reflecting an evolution from reactive measures to proactive, adaptive, and autonomous defence strategies. ^70^ Overall, the publication patterns demonstrate both quantitative growth and thematic diversification, with research increasingly intersecting with governance, ethics, and cross-domain resilience. Figure 4 visualizes the growth of literature related to Agentic AI in cybersecurity across academic, industrial, and policy domains from 2005 to 2025. This temporal trend highlights a sharp increase in activity post-2015, coinciding with the rise of autonomous AI systems and growing quantum-era security concerns.

### 3.4 Geographical distribution - Mapping global research activity and drivers

Research into agentic AI in cybersecurity shows a globally diverse landscape, with notable activity in the United States, India, the United Kingdom, and China. These regions lead in publications and innovation, driven by national security priorities, technology sector investment, and advanced academic research ecosystems. ^71^ Strategic adoption of AI in cybersecurity is further evident in regions investing in national resilience, such as the integration of AI-enhanced infrastructure protection in smart grids, transportation, and crisis management systems. Countries with mature digital economies, including the US, Singapore, and parts of the EU, are increasingly embedding AI into multi-sectoral cybersecurity strategies. ^72^ The geographical spread also reflects emerging markets’ growing role in AI-driven cybersecurity research. Nations such as the UAE are focusing on adoption readiness, with socio-cultural and workforce factors shaping implementation strategies. ^73^ Finally, global distribution trends indicate increasing collaboration between AI hubs and regions with sector-specific vulnerabilities such as healthcare, logistics, and energy through cross-border research initiatives and targeted deployments. ^74^

**Figure 5.**
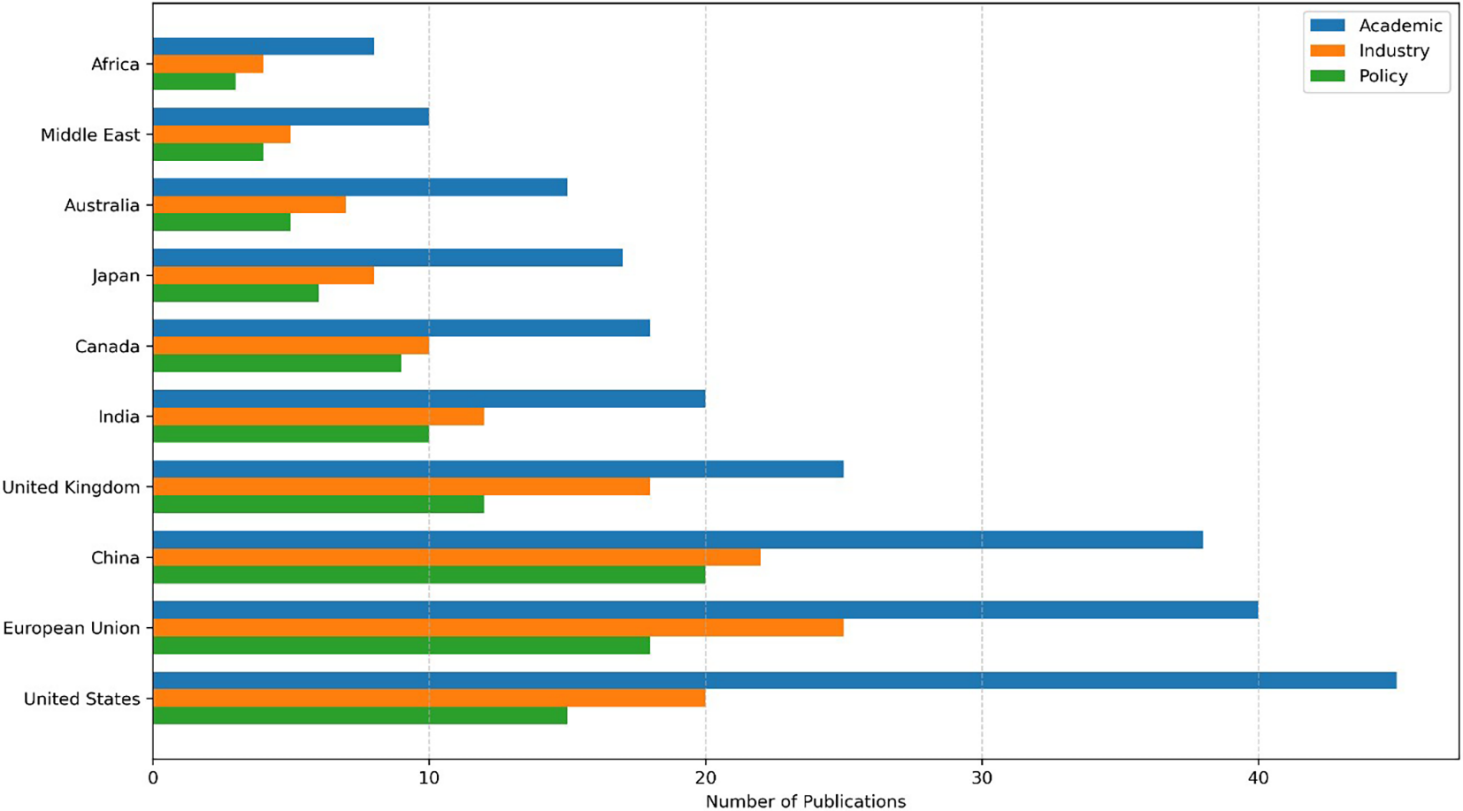
Geographical distribution of research.

While research activity is concentrated in regions such as North America, Western Europe, and parts of East Asia, the geographical distribution also reveals notable gaps. Africa, Latin America, and parts of Southeast Asia are significantly underrepresented in the reviewed literature. This disparity suggests the need for more inclusive global participation in developing and governing Agentic AI systems for cybersecurity, especially in regions with rising digital infrastructure but limited AI policy maturity. Figure 5 presents a comparative analysis of research activity across global regions, highlighting regional leadership in agentic AI for cybersecurity. The United States, the EU, and China dominate academic and policy-related outputs, with emerging contributions from the Middle East and Africa.

### 3.5 Technology taxonomy - Categorization by autonomy level, domain, governance type

The technology taxonomy for agentic AI in cybersecurity can be structured along three key dimensions: autonomy level, application domain, and governance type.

Autonomy Level: Agentic AI systems range from semi-autonomous assistants that require human-in-the-loop oversight to fully autonomous agents capable of adaptive decision-making in dynamic cyber environments. Händler ^75^ proposes a multi-dimensional taxonomy that evaluates autonomy across aspects such as task management, agent collaboration, and context interaction, emphasizing the balance between independence and alignment. Cihon et al. ^76^ further refine autonomy assessment through a code-based inspection method that measures agent independence and required oversight.

Application Domain: Agentic AI applications in cybersecurity span critical infrastructure defense, malware detection, threat intelligence, blockchain security, and autonomous incident response. Karim et al. ^77^ highlight blockchain-integrated multi-agent systems for secure and scalable collaboration in decentralized environments, demonstrating interoperability across sectors such as finance, Web3, and autonomous systems.

Governance Type: Governance structures for agentic AI can be centralized, decentralized, or hybrid. Frenette ^78^ introduces Decentralized AI Governance Networks (DAGN) with tokenized power control to enforce human-centric policies, particularly relevant for sensitive domains like cybersecurity. Similarly, the LOKA Protocol provides a governance-oriented architecture with decentralized identity and ethical consensus mechanisms to ensure trustworthy multi-agent operations. ^79^

**Figure 6.**
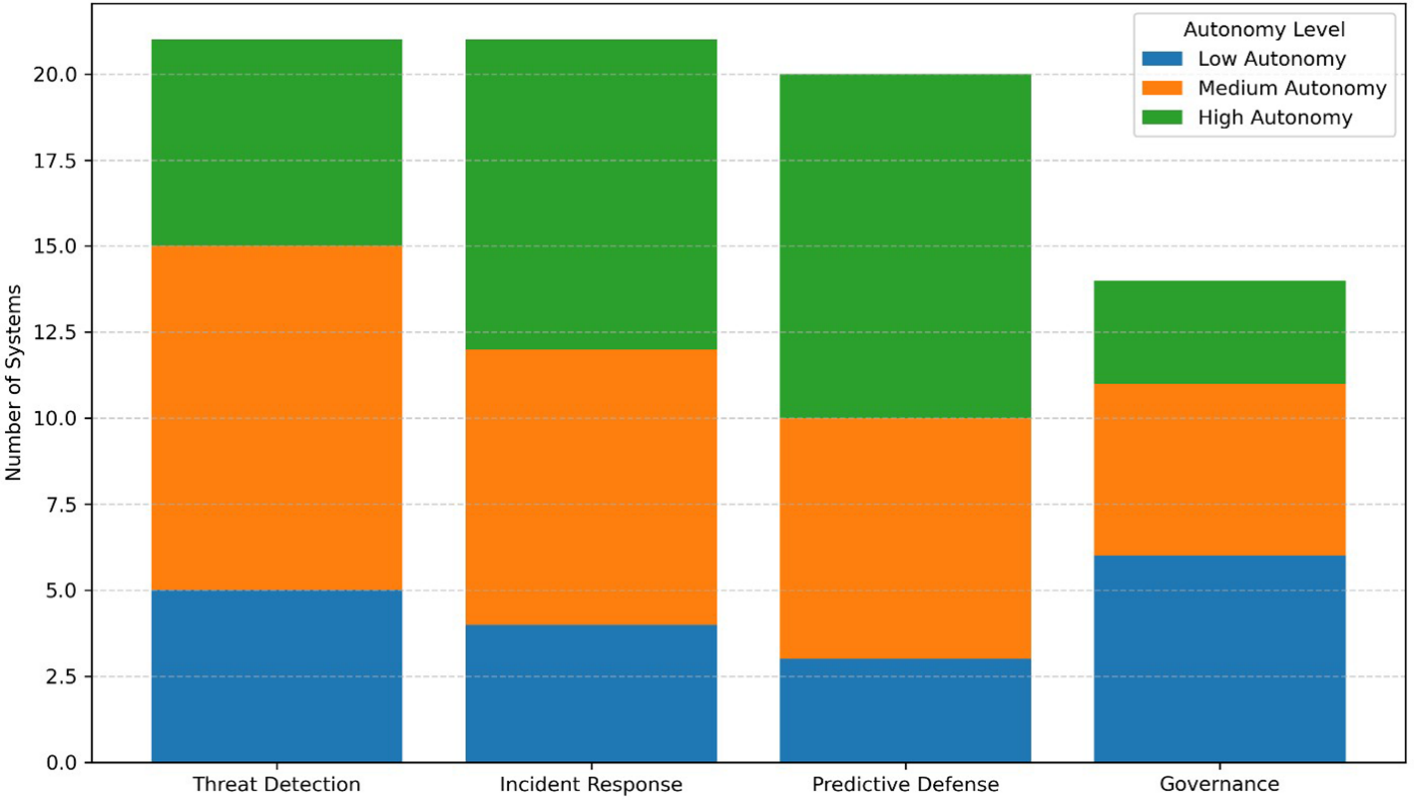
Technology taxonomy.

This taxonomy framework supports the systematic classification of agentic AI systems, enabling researchers and practitioners to evaluate trade-offs between operational autonomy, security domain applicability, and governance robustness. Figure 6 presents a structured taxonomy of agentic AI technologies in cybersecurity, categorizing them by autonomy levels, operational domains, and governance models. This visual classification highlights the diversity of system configurations and control mechanisms in real-world and experimental deployments.

## 4. Findings

### 4.1 Overview of literature trends

**Figure 7.**
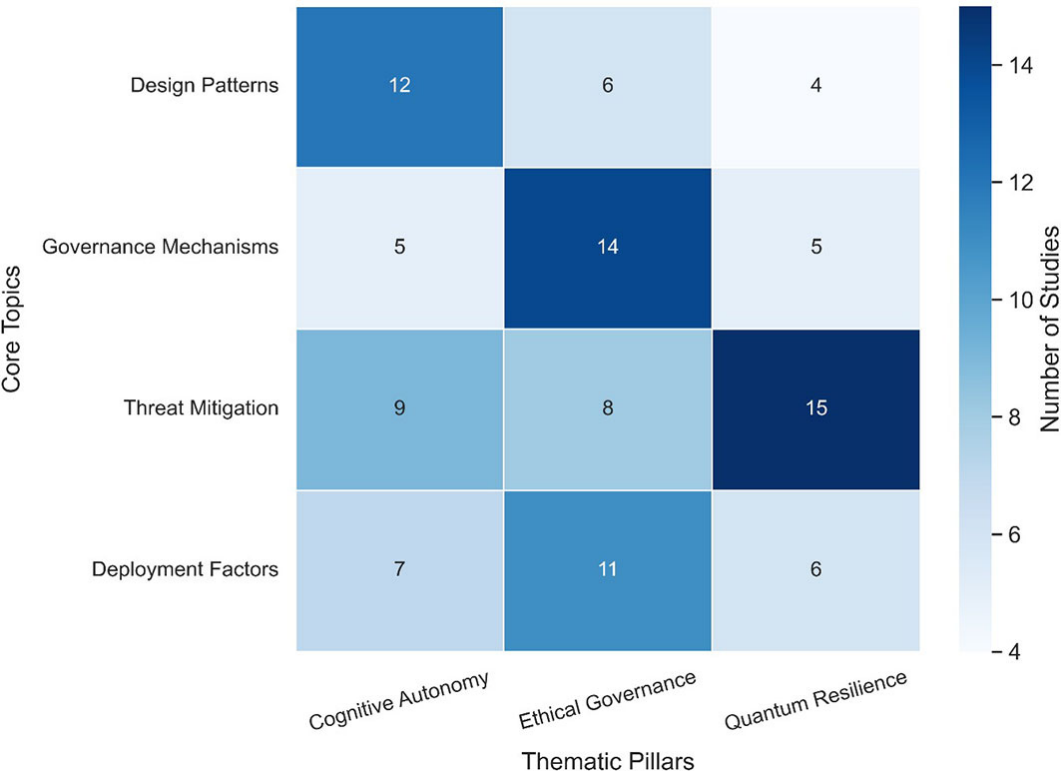
Cross-pillar evidence matrix - mapping reviewed works against thematic pillars.

The literature on agentic AI in cybersecurity demonstrates a steady trajectory toward maturity, reflected in publication growth, thematic consolidation, and expanding international contributions. A recent bibliometric analysis of agent-based systems in cybersecurity revealed a consistent upward trend since 2013, with a marked acceleration after 2018. The field reached a peak in 2023 with over 1,200 publications and more than 30,000 citations, indicating both academic interest and practical relevance. ^68^ Geographically, China emerged as the largest contributor, followed closely by the United States and India, reflecting both state-led research investment and private sector innovation. The thematic landscape is dominated by intrusion detection, malware classification, blockchain security, and emerging quantum-resilient methods. ^71^ Moreover, the convergence of agentic AI with adjacent domains such as blockchain and federated learning illustrates the transition from experimental prototypes to scalable, operational systems. This shift parallels a move toward multi-stakeholder collaborations and increased cross-border research networks, which are characteristic indicators of a maturing research field. ^69^ These patterns suggest that agentic AI in cybersecurity is entering an applied innovation phase, where theoretical development is increasingly complemented by field deployment and governance considerations. Figure 7 synthesizes reviewed studies across the three thematic pillars of this review: Cognitive Autonomy, Ethical Governance, and Quantum Resilience, using a cross-pillar evidence matrix. The heatmap highlights topic -pillar intersections and identifies underexplored thematic areas in agentic AI research.

RQ1: Common Design Patterns in Agentic AI Cybersecurity

Agentic AI in cybersecurity often leverages hybrid architectures that integrate symbolic reasoning with data-driven methods to improve adaptability and interpretability. For example, hybrid AI cyber defense agents have been developed combining deep reinforcement learning (DRL), large language models (LLMs), and rule-based reasoning for real-time threat detection, network monitoring, and human -agent collaboration. ^80^ Architectural design patterns for agentic AI frequently follow modular frameworks where components such as perception, reasoning, and action layers are clearly separated and can be combined dynamically. Taxonomies like the boxology approach extend to hybrid actor systems, enabling distributed reasoning and coordinated multi-agent operations. ^81– 83^ Explainability has emerged as a critical design pillar in these systems, with patterns such as TriQPAN (Trigger, Query, Process, Action, Notify) embedding explainability into the agent’s decision loop, thereby enhancing trust in autonomous operations. ^84^ Hybrid human -agent teaming patterns, such as delegation and associative teaming, structure interactions to optimize cognitive load distribution between human operators and AI agents, especially in high-stakes cybersecurity operations. ^85^ Table 2 outlines recurring design patterns identified in the literature for agentic AI systems used in cybersecurity. These patterns include architectural models, decision-making approaches, and integration strategies with human or hybrid systems. Understanding these design archetypes provides a foundation for assessing both operational strengths and vulnerabilities in emerging agentic defense frameworks.

**Table 2.** Common design patterns with examples.

Design pattern	Description	Example application	Autonomy level
Reactive Agents	Rule-based responses to predefined threats	Signature-based malware detection	Low
Proactive Goal-Seeking	Agents that plan actions based on objectives and environmental scanning	Adaptive firewall reconfiguration	Medium
Learning-Based Agents	Agents trained via ML to detect evolving threats	Anomaly detection using reinforcement learning	High
Human-in-the-Loop	The agent acts autonomously but escalates to a human under uncertainty	Threat triage assistants in SOCs	Medium
Federated Agent Networks	Distributed agents learning from local data without central sharing	Federated malware classifiers across orgs	Medium -High
Reflexive Cognitive Loops	Agents with internal reasoning about confidence, ethics, or system feedback	Self-regulating AI-based honeypots	High

Case study - Autonomous threat detection system:

A deployed system integrating DRL-driven agents with LLM-based analyst interfaces demonstrated robust performance in defending critical networks against simulated red-team attacks. The system dynamically selected between monitoring, deception, and remediation strategies, outperforming baseline static defenses in adversarial testing environments. ^80^

RQ2: Ethical Governance Mechanisms and Compliance Alignment

The governance of Agentic AI in cybersecurity requires integrating ethical oversight, regulatory compliance, and operational best practices into system design and deployment. Frameworks such as the NIST AI Risk Management Framework (AI RMF), ISO/IEC AI standards, and the EU AI Act have emerged as central pillars for ensuring trustworthiness, safety, and accountability in AI-driven defense systems. These frameworks aim to harmonize technical controls with societal expectations by embedding fairness, transparency, and risk-based assessment throughout the AI lifecycle. ISO/IEC Standards, notably ISO/IEC 42001:2023, provide a certifiable management system for AI governance, outlining requirements for risk assessment, accountability structures, and continuous auditing processes. This standard is particularly relevant for cybersecurity applications, as it introduces operational guidance for AI risk mitigation and conformity assessment, helping organizations align with international compliance requirements. ^86^ The EU AI Act adopts a risk-based approach to AI regulation, classifying systems into risk categories and enforcing stricter governance for high-risk applications such as autonomous cybersecurity agents. Its provisions mandate transparency, human oversight, and conformity with fundamental rights, while also fostering cross-border regulatory harmonization. ^87^ Complementing this, the EU’s Ethics Guidelines for Trustworthy AI advocate seven key requirements, including technical robustness, accountability, and privacy protection, offering a non-binding yet influential blueprint for ethical AI deployment. ^88^ In the U.S., the NIST AI RMF emphasizes risk identification, mitigation strategies, and measurement of trustworthiness metrics. When combined with ISO/IEC standards, it enables interoperability between domestic and international governance regimes, reducing compliance fragmentation and ensuring AI systems meet both security and ethical imperatives. ^89^ Comparative analyses show that while ISO standards excel in operational consistency, they often lack enforcement mechanisms, making legally binding instruments like the EU AI Act critical for ensuring adherence in sensitive cybersecurity contexts. ^90^ Moving forward, integrating human rights-based governance approaches could enhance the ethical legitimacy of agentic AI, particularly in scenarios involving autonomous decision-making in cyber defense. ^91^ Table 3 compares leading governance frameworks relevant to agentic AI systems in cybersecurity. The table highlights each framework’s core principles, scope of applicability, and alignment with ethical requirements such as transparency, accountability, and human oversight. This synthesis offers a practical reference point for evaluating governance integration within agentic AI deployments.

**Table 3.** Governance approaches and compliance alignment.

Governance framework	Core focus areas	Scope of applicability	Ethical alignment areas
NIST AI RMF (USA)	Risk management, trustworthiness	Federal and private sector (USA)	Fairness, explainability, accountability
EU AI Act (EU)	Risk-based AI classification	High-risk systems in EU jurisdictions	Human oversight, safety, transparency
ISO/IEC 42001	AI management systems and lifecycle	Global, industry-agnostic	Governance, responsibility, documentation
IEEE 7000 Series	Design ethics and value alignment	System design and implementation	Value alignment, stakeholder engagement
OECD AI Principles	Global normative guidance	Multilateral, public-private sectors	Robustness, democratic values, and human rights
UNESCO AI Ethics	Cross-cultural ethical foundations	Global education, human development	Sustainability, cultural diversity, and inclusion

**Figure 8.**
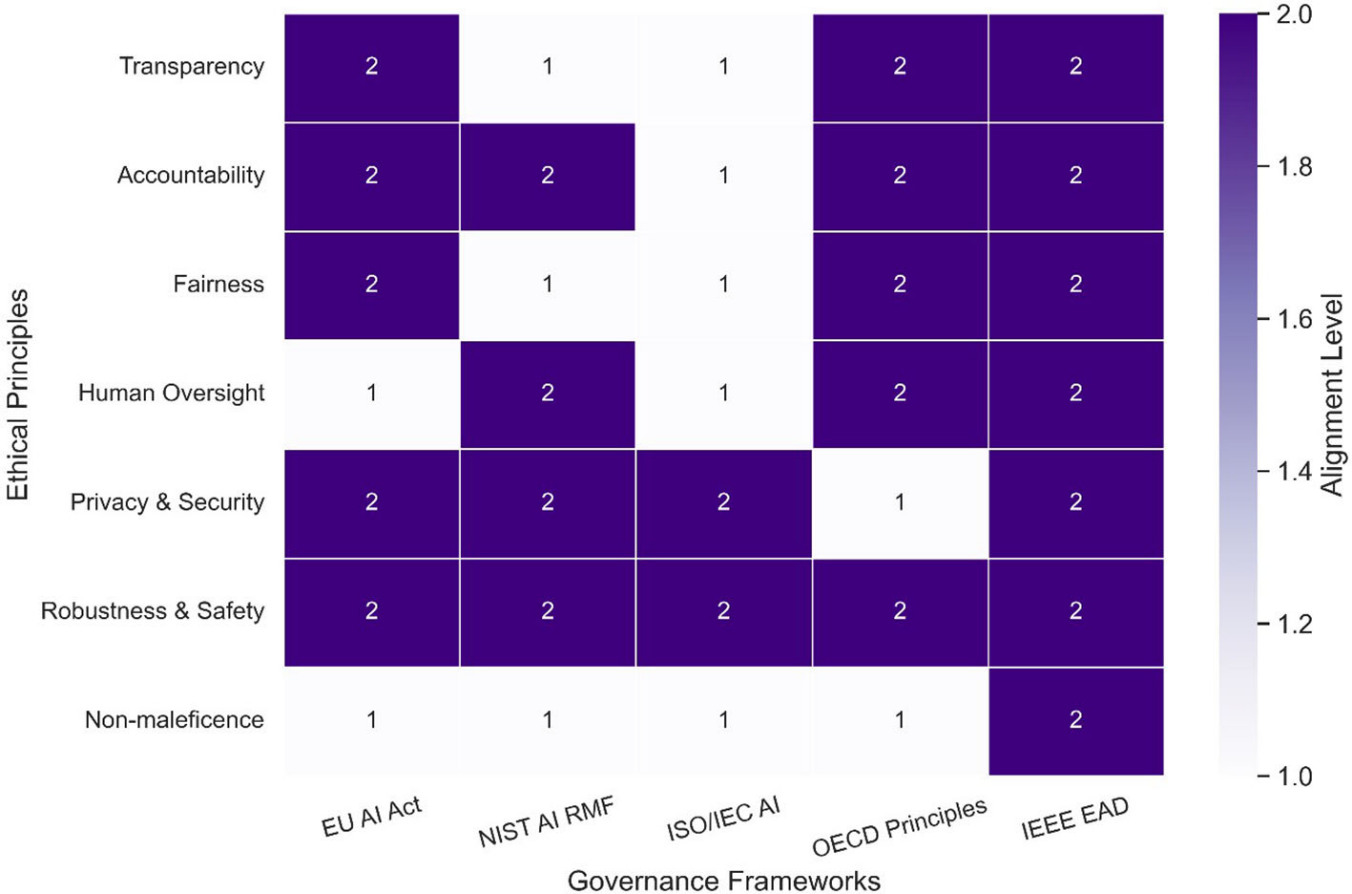
Governance - ethical principle mapping.

Figure 8 maps the alignment between leading AI governance frameworks and foundational ethical principles, offering a comparative visual of strengths, overlaps, and potential gaps. By illustrating how ethical concerns like transparency, accountability, and fairness are addressed (or omitted) across frameworks such as the EU AI Act, NIST AI RMF, and ISO/IEC standards, the figure clarifies areas of convergence and fragmentation within agentic AI governance.

RQ3: Threat Resilience Strategies (Conventional & Quantum)

Cybersecurity resilience strategies must now address both conventional threats and the disruptive potential of quantum computing. Traditional approaches such as layered defense architectures, intrusion detection, and zero-trust models remain crucial but increasingly integrate AI-driven predictive analytics for proactive risk mitigation. ^92^ Quantum resilience demands post-quantum cryptography (PQC) to safeguard against algorithms like Shor’s, which threaten RSA and ECC encryption. Lattice-based, hash-based, and multivariate schemes are emerging as leading candidates, often paired with AI for adaptive key management. ^93^ Hybrid frameworks that combine traditional and quantum-resistant algorithms ensure a gradual migration path while maintaining operational continuity. ^94^ Beyond cryptography, quantum-safe strategies focus on AI model integrity, addressing risks such as adversarial inputs and data poisoning through deep-learning-based anomaly detection and graph neural networks. ^95^ Quantum-secure threat intelligence platforms that leverage generative AI for predictive modeling and integrate PQC provide long-term resilience against evolving threats. ^96^ Table 4 presents a comparative overview of threat resilience strategies employed in agentic AI systems, differentiating between conventional cybersecurity defenses and quantum-resilient approaches. By mapping specific techniques, use cases, and implementation readiness levels, this table provides a structured perspective on how agentic systems are evolving to address both present and emerging threat vectors, particularly in the context of post-quantum risk environments.

**Table 4.** Comparative overview of threat resilience strategies.

Strategy type	Technique/Approach	Example use case	Implementation readiness
Conventional Defense	Adversarial training	Robust anomaly detection	Mature
Conventional Defense	Multi-agent redundancy	Fail-safe decision-making agents	Intermediate
Conventional Defense	Secure model update pipelines	Tamper-resistant agent learning	Intermediate
Quantum-Resilient	Post-quantum cryptography (PQC)	Agent communication using lattice cryptography	Emerging
Quantum-Resilient	Quantum key distribution (QKD)	Secure multi-agent key exchange	Experimental
Quantum-Resilient	Quantum-safe federated learning	Distributed anomaly detection with PQC	Emerging

**Figure 9.**
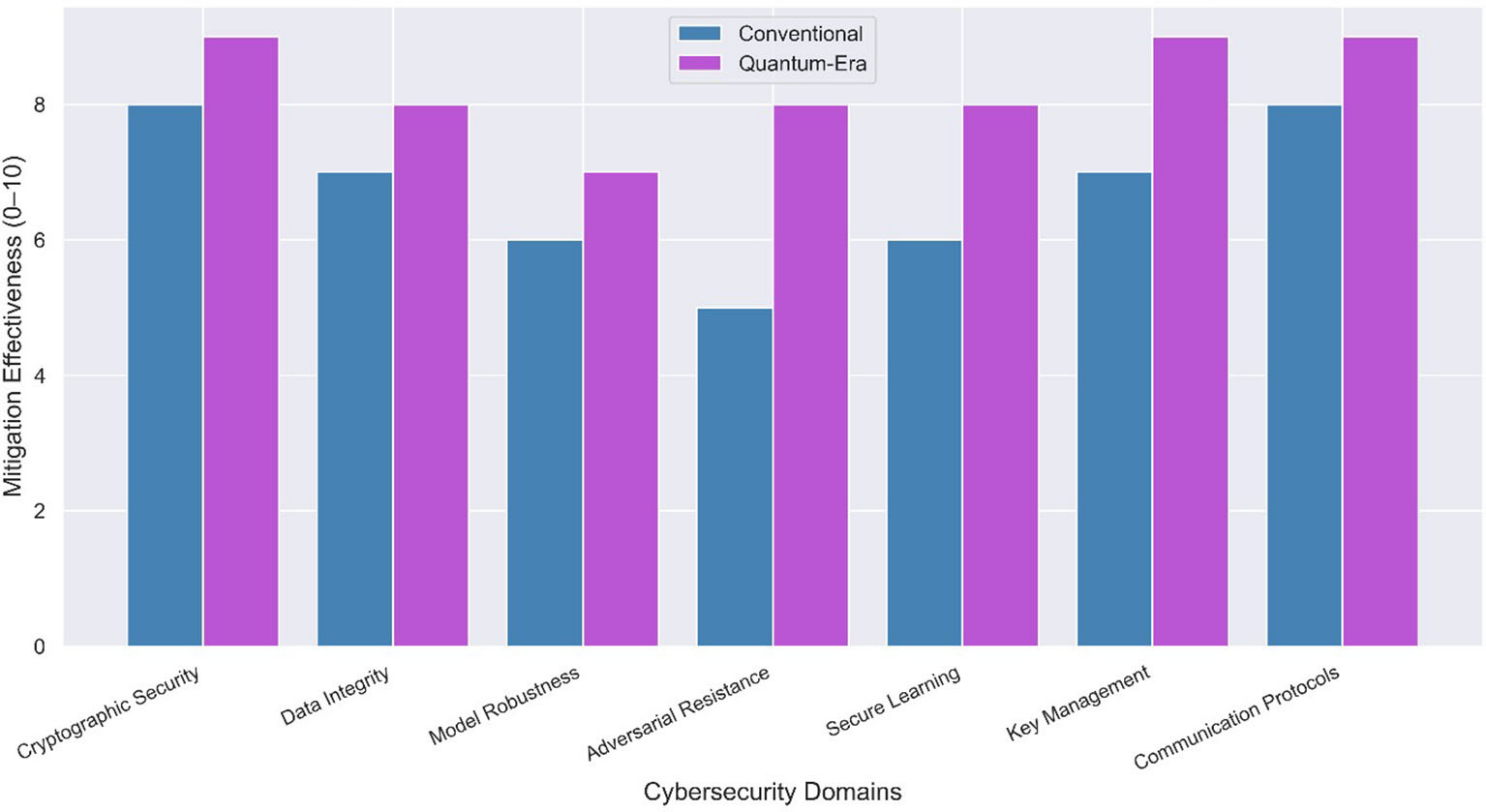
Side-by-side mapping of conventional vs quantum readiness.

Figure 9 contrasts conventional and quantum-era threat mitigation strategies across core cybersecurity domains. The comparative bar chart illustrates how mitigation approaches evolve in response to emerging quantum threats, highlighting both continuity and strategic shifts in defense postures for agentic AI systems.

Case study: In a recent quantum-safe AI pilot, a hybrid architecture was deployed in a multi-cloud environment, integrating Kyber and McEliece PQC algorithms with real-time AI-driven key management. The system demonstrated sub-4ms encryption latency while achieving zero vulnerability to Shor’s algorithm and minimal susceptibility to Grover’s. ^97^ Combining conventional defenses with AI-enhanced quantum-safe mechanisms, especially hybrid cryptographic models and predictive threat intelligence, offers the most effective path to achieving future-proof resilience against both current and quantum-enabled cyber threats.

RQ4: Implementation Barriers and Enablers

The deployment of agentic AI in cybersecurity is shaped by a complex interplay of barriers and enablers that determine scalability, trust, and operational success. Common barriers include high implementation costs, integration complexity with legacy systems, and a shortage of skilled professionals capable of designing, deploying, and maintaining multi-agent AI architectures. Organizational resistance to change and ethical concerns, such as data privacy and algorithmic bias, further slow adoption. ^98^ In military and critical infrastructure contexts, additional hurdles include ensuring model robustness under adversarial conditions and safeguarding sensitive operational data during training and deployment. ^99^ On the enabling side, strong governance frameworks, cross-sector collaboration, and investment in workforce training are critical. For example, secure multi-agent communication protocols such as Google’s Agent2Agent (A2A) and Model Context Protocol (MCP) provide a robust foundation for interoperability and resilience in complex agent ecosystems. ^100^ Multi-stakeholder collaboration where government, academia, and industry jointly participate in development and oversight has been shown to accelerate safe deployment while improving compliance with security and ethical guidelines. ^101^ Table 5 synthesizes key implementation barriers and enabling factors influencing the deployment of agentic AI systems in cybersecurity contexts. Drawing from both academic and grey literature, this table captures the technical, organizational, and regulatory dynamics that affect real-world adoption, offering insights into how these systems can be scaled effectively and ethically across diverse operational environments.

**Table 5.** Summary of barriers and enablers.

Category	Barrier	Enabler
Technical	Model complexity and interpretability	Modular architectures and explainable AI (XAI)
Human Capital	Skills gap in secure agentic AI design	Cross-sector training and capacity-building
Organizational	Integration with legacy infrastructure	Cloud-native deployment models
Regulatory	Ambiguous compliance obligations	Aligned ethical governance frameworks
Economic	High cost of development and maintenance	Public-private funding mechanisms
Interoperability	Lack of standards across systems	Open protocol ecosystems and industry coalitions

A successful example of overcoming barriers can be seen in large-scale, human-centered deployment frameworks in healthcare AI, which emphasize modular architecture, explainability, and continuous monitoring to maintain operational trust principles equally applicable to cybersecurity agentic AI systems. ^102^ These approaches demonstrate that the combination of strong technical safeguards, inclusive governance, and adaptive organizational culture is key to unlocking the full potential of agentic AI in cybersecurity.

## 5. Discussion

### 5.1 Summary of key insights

The integration of Agentic AI into cybersecurity frameworks reveals a convergence of four core dimensions: design patterns, governance mechanisms, resilience strategies, and implementation dynamics that collectively define the field’s maturity. From a design perspective, agentic architectures are increasingly adopting reusable patterns that enhance scalability, safety, and multi-agent coordination, enabling systems to autonomously manage complex cybersecurity tasks while maintaining human oversight where necessary. ^103^ The shift toward modular control planes, interoperable tool orchestration, and context-aware decision-making has been critical for adapting to diverse operational environments. On the governance front, alignment with global frameworks such as the NIST AI RMF and sector-specific compliance models has proven essential to ensuring transparency, ethical accountability, and lawful operation in automated threat response systems. ^104^ These governance measures are further strengthened by privacy-by-design principles, which mitigate risks of misuse and enhance stakeholder trust. In terms of resilience strategies, advancements have moved beyond conventional cryptography toward quantum-resilient AI systems capable of maintaining operational integrity even against post-quantum adversaries. ^63^ This includes integrating anomaly detection, autonomous incident response, and adaptive behavioral analytics to counter rapidly evolving threats. Finally, implementation success is closely linked to collaboration between stakeholders, availability of skilled talent, and sustainable funding models. Case studies show that multi-stakeholder deployments combining technical, policy, and operational expertise are more effective at achieving both security and compliance goals. ^105^ However, persistent challenges remain in integrating these systems into legacy infrastructures without introducing operational complexity or excessive cost burdens. Overall, the field’s trajectory suggests that Agentic AI in cybersecurity is moving toward a mature ecosystem where autonomous defense capabilities are harmonized with robust governance and quantum-ready resilience, creating a dynamic yet ethically anchored security posture.

### 5.2 Comparison with existing reviews

Previous literature on AI in cybersecurity has largely concentrated on either the technical mechanisms of AI-driven defense or high-level discussions of AI ethics, with relatively few works explicitly examining the convergence of agentic autonomy, governance integration, and quantum-resilient defense. For instance, many traditional reviews, such as Daraojimba et al., ^106^ provide comprehensive overviews of AI applications in protecting national infrastructure but lack an in-depth exploration of how agentic architectures adaptively align with evolving governance frameworks and resilience strategies. Similarly, while Tallam ^63^ advances the discussion by integrating ethical governance into AI-driven cyber defense, its focus remains on operational frameworks rather than the multi-pillar synthesis of design patterns, governance, and post-quantum security that our review aims to deliver. Some reviews, such as Al Siam et al., ^107^ offer a holistic analysis of AI in cybersecurity, categorizing technical advances across domains like threat detection, endpoint protection, and adaptive authentication. However, they typically treat governance and quantum readiness as peripheral topics rather than integral co-drivers of system maturity. Furthermore, Oesch et al. ^64^ explore agentic AI in the context of cyber conflict and global security competition but do not address operational barriers, stakeholder collaboration mechanisms, or the integration of emerging resilience strategies into national cybersecurity postures. By contrast, this review extends beyond these prior works by explicitly mapping design patterns to governance compliance and resilience measures, synthesizing both conventional and quantum-era defense considerations. It also draws on cross-domain analogies and empirical case studies to bridge the gap between theory and implementation, offering a multi-pillar framework for understanding and advancing the role of Agentic AI in cybersecurity.

### 5.3 Research trends & gaps

Recent studies reveal that research on agentic AI in cybersecurity has accelerated, particularly in areas such as intrusion detection, malware classification, and IoT security, with emerging interest in adversarial machine learning, blockchain integration, and quantum-resilient approaches. ^71^ Bibliometric analyses show steady growth in publications since 2013, with 2023 marking a peak in both research output and citations, underscoring the expanding global attention to agent-based cybersecurity systems. ^68^ Several thematic trends are emerging. First, AI-powered threat intelligence and anomaly detection are becoming standard components of advanced cyber defense systems. ^108^ Second, research increasingly explores multi-agent architectures for cross-domain knowledge discovery, enhancing the adaptability and contextual reasoning of AI-driven security platforms. ^109^ Third, workforce capability gaps remain a pressing issue, with global demand for AI skills in cybersecurity, such as predictive analytics and neural networks, outpacing current talent supply. ^110^ Despite these advances, significant gaps persist. Conceptual research still dominates over large-scale empirical deployments, limiting real-world validation of agentic AI’s resilience under adversarial conditions. ^111^ Ethical governance and compliance integration, while discussed often, remain underdeveloped in practice, with few frameworks aligning technical safeguards to emerging regulatory mandates. ^112^ Additionally, the integration of quantum-safe AI remains largely at the pilot stage, with limited cross-sectoral adoption or testing in high-threat operational environments. ^70^ While agentic AI research in cybersecurity is rapidly expanding with promising innovations, there is a critical need for empirical validation, workforce capability building, ethical governance alignment, and proactive readiness for quantum-era threats. Table 6 identifies critical research gaps uncovered through this review and proposes corresponding future research questions. These gaps span across technical innovation, ethical governance, and resilience to emerging threats. The questions are intended to guide scholars, practitioners, and policymakers in shaping the next wave of research on agentic AI in cybersecurity.

**Table 6.** Gaps and future priorities.

Thematic area	Identified gap	Future research question
Cognitive Autonomy	Lack of standardization in agent reasoning models	How can we formalize and benchmark cognitive autonomy in AI agents for cybersecurity?
Ethical Governance	Limited empirical work on applied dual-use mitigation	What practical governance tools can prevent misuse of defensive agentic AI systems?
Quantum-Resilient Design	Early-stage exploration of quantum-safe federated learning	How can federated agentic AI systems ensure resilience to quantum-enabled threats?
Socio-Technical Systems	Under-researched human -agent trust calibration	What design strategies enhance human trust in semi-autonomous defense agents?
Compliance Integration	Fragmented alignment between technical and regulatory systems	How can agentic AI architectures be made auditable and legally interpretable?
Cross-Domain Insights	Lack of translatable insights from other high-stakes domains	What lessons from aviation or healthcare can inform safe agentic AI deployment in cybersecurity?

### 5.4 Practical implications

The integration of Agentic AI into cybersecurity presents a set of actionable pathways for stakeholders, including policymakers, developers, and security managers, to enhance resilience and trust in autonomous defense systems. For policymakers, there is a pressing need to establish regulatory frameworks that balance innovation with public safety. This includes ensuring transparency, enforcing ethical use standards, and aligning governance with internationally recognized frameworks such as the NIST AI Risk Management Framework and the EU AI Act to mitigate misuse and protect civil liberties. ^113^ For developers, secure-by-design principles should be prioritized to address vulnerabilities inherent in agentic architectures, such as data poisoning, adversarial manipulation, and unauthorized access. Approaches such as the MAESTRO risk framework for Agent-to-Agent (A2A) protocols can guide the development of resilient and interoperable systems. ^114^ Furthermore, explainable AI (XAI) techniques should be embedded to support monitoring, auditing, and compliance with ethical norms. ^115^ Security managers must adapt operational models to leverage Agentic AI’s predictive and adaptive capabilities while maintaining human oversight for high-stakes decision-making. Implementing hybrid human -agent workflows can enhance detection and response without fully relinquishing control, especially in critical infrastructure defense. Collaboration across sectors is essential for sharing threat intelligence and developing quantum-resilient strategies that protect AI models and cryptographic assets from emerging quantum computing threats. ^116^ Ultimately, the practical application of Agentic AI in cybersecurity hinges on a triad of well-aligned governance, secure system design, and proactive operational strategies. When these elements are integrated, Agentic AI can serve as a force multiplier for defense capabilities while upholding the principles of accountability, transparency, and ethical stewardship.

### 5.5 Ethical & policy considerations

The integration of agentic AI into cybersecurity systems presents complex ethical and policy challenges that require careful governance. Central concerns include transparency, accountability, legal compliance, and dual-use governance. Transparency is critical for fostering trust, ensuring that AI decision-making processes remain explainable and open to scrutiny, particularly in high-stakes security contexts. ^117^ Accountability frameworks must address the difficulty of attributing responsibility in autonomous systems, incorporating auditability and clear chains of responsibility. ^118^ Legal compliance is also essential, as AI-driven cybersecurity must align with data protection regulations such as GDPR and evolving AI-specific governance standards. ^104^ In addition, the dual-use nature of AI technologies, where tools designed for defense could be repurposed for offensive cyber operations, necessitates proactive policy safeguards to mitigate misuse. ^119^ Ethical governance models should integrate fairness-aware AI design, bias mitigation strategies, and public engagement mechanisms to align technological deployment with societal values. ^120^ Ultimately, effective policy must balance innovation with human rights protections, ensuring that agentic AI in cybersecurity operates within robust ethical and legal boundaries.

### 5.6 Strengths of this review

This review distinguishes itself through its breadth, diversity, and integrative approach, enabling a holistic understanding of agentic AI in cybersecurity. Unlike narrowly focused studies, narrative reviews can accommodate a wide range of sources, synthesize cross-disciplinary perspectives, and highlight both conceptual and empirical developments in the field. ^121^ This methodological flexibility allows for the inclusion of technological, ethical, and governance dimensions, which are crucial in an area as multifaceted as cybersecurity enhanced by AI. The diversity of literature integrated here, spanning technical frameworks, governance models, and sector-specific applications, enables a richer contextualization of findings, aligning with best practices in high-quality narrative synthesis. ^122^ Furthermore, this work extends beyond descriptive aggregation by critically linking patterns across governance, threat resilience, and implementation strategies, thereby offering an actionable synthesis for policymakers, developers, and security managers. Finally, the integration of multi-sector insights mirrors the strengths highlighted in previous interdisciplinary narrative reviews, which have demonstrated the value of cross-pollination between fields to address complex socio-technical challenges. ^123^ This review’s ability to weave together perspectives from different disciplines ensures that its conclusions are robust, relevant, and adaptable to rapidly evolving technological landscapes.

### 5.7 Future research directions

Future research on agentic AI in cybersecurity should be structured across short-, medium-, and long-term horizons, with each phase addressing pressing challenges and laying the groundwork for more advanced solutions. In the short term (1-3 years), emphasis should be placed on enhancing XAI and robust adversarial defense mechanisms to counteract model poisoning, data manipulation, and zero-day vulnerabilities. Recent studies have shown that while AI-driven anomaly detection and threat prediction significantly improve incident response, their susceptibility to adversarial attacks remains a key obstacle to operational deployment. ^70^ In the medium term (3-7 years), the integration of federated learning with quantum-safe cryptographic protocols is expected to gain prominence. This approach can enable decentralized model training without exposing sensitive data, while resisting the decryption capabilities of quantum computers. Research in cyber-physical systems security highlights the potential of combining AI, blockchain, and quantum-resistant algorithms to create scalable, privacy-preserving defense frameworks. ^124^ In the long term (7+ years), the focus will likely shift toward neurosymbolic AI and quantum-enhanced multi-agent reinforcement learning for autonomous and adaptive threat mitigation. Neurosymbolic AI promises enhanced reasoning and explainability by combining symbolic knowledge graphs with deep learning models, making AI systems more transparent and reliable in high-stakes security environments. ^125^ Concurrently, quantum multi-agent reinforcement learning could enable faster, more coordinated responses to cyber incidents, leveraging quantum computational speedups for complex decision-making. ^126^

An emerging technology watchlist should therefore include:

• Neurosymbolic AI for explainable and safe decision-making in cybersecurity.
• Federated learning integrated with quantum-safe protocols for secure, decentralized intelligence.
• Quantum-enhanced AI architectures for rapid, scalable, and autonomous security orchestration.

By strategically aligning research with these phased horizons, the cybersecurity field can evolve toward highly autonomous, explainable, and quantum-resilient agentic AI systems capable of operating securely in complex, adversarial digital ecosystems.

### 5.8 Limitations of the review

While this review provides a broad and integrative synthesis of agentic AI in cybersecurity, several limitations should be acknowledged. First, the scope is inherently constrained by the availability and accessibility of relevant literature, which may introduce selection bias. Narrative and systematic reviews in AI research often face challenges in ensuring full representativeness of the field, particularly when grey literature and non-English sources are excluded, potentially limiting generalizability to global contexts. ^127^ Second, while the methodology applied aimed for rigor, the reliance on human interpretation during thematic synthesis may introduce subjectivity, a recognized limitation in narrative synthesis approaches. ^128^ In addition, differences in study design, evaluation metrics, and reporting standards across included works make comparative analysis challenging, potentially affecting the reliability of cross-study conclusions. ^129^ Third, this review’s findings may be influenced by the publication bias prevalent in AI and cybersecurity literature, where studies reporting positive or novel outcomes are more likely to be published than those reporting null or negative results. ^130^ Finally, given the rapid evolution of AI technologies, there is an inherent temporal limitation; insights drawn from current literature may become outdated as breakthroughs and regulatory changes emerge. ^131^ While the synthesis offers valuable integrative insights, these methodological and contextual constraints should be considered when interpreting the results and their applicability to future research or policy contexts.

### 5.9 Cross-domain insights

Lessons from other critical industries aviation, finance, and healthcare, offer valuable parallels for agentic AI in cybersecurity. In aviation, AI-driven anomaly detection, predictive analytics, and game theory -based adversarial modeling have been successfully deployed for avionics security, airport monitoring, and autonomous flight operations. These approaches emphasize the importance of certified, trustworthy AI solutions within highly regulated environments, ensuring both operational safety and regulatory compliance. ^132^ In the financial sector, cross-domain data sharing, particularly in green finance, has demonstrated how AI can integrate diverse datasets across organizational boundaries to enhance decision-making and compliance monitoring. This model underscores the potential for agentic AI to facilitate secure, privacy-preserving data exchanges in cybersecurity contexts. ^133^ In healthcare, the value of multi-agent AI systems for interdisciplinary collaboration offers a template for cybersecurity. Multi-AI frameworks have been shown to enhance knowledge integration, decision-making speed, and contextual adaptability, especially when dealing with complex, multi-variable scenarios. ^109^ Taken together, these cross-domain experiences highlight three transferable principles: robust certification and governance of AI tools, secure and ethical cross-organization data sharing, and collaborative multi-agent ecosystems capable of adapting to high-stakes, dynamic environments. Cross-domain insights from aviation, finance, and healthcare provide valuable lessons for agentic AI in cybersecurity. In aviation, autonomous flight control systems, such as Airbus’s AI-enabled Flight Management Systems, illustrate how machine agency can be deployed in high-stakes, safety-critical environments with rigorous redundancy, explainability, and human override protocols. In finance, AI-driven fraud detection systems such as Mastercard’s Decision Intelligence combine cognitive autonomy with compliance-by-design architectures, offering models for integrating ethical governance into algorithmic decision-making. These examples underscore the importance of embedding trust, accountability, and resilience into agentic systems, especially when scaling into volatile cybersecurity domains.

### 5.10 Analytical framework of agentic AI in cybersecurity

**Figure 10.**
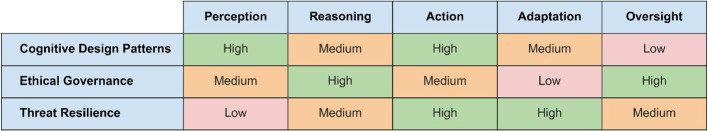
Integrated analytical framework (Design -Governance -Resilience).

An effective analytical framework for agentic AI in cybersecurity integrates design principles, governance structures, and resilience mechanisms into a unified model. At its core, the framework must incorporate multi-layered threat intelligence pipelines, real-time anomaly detection, automated response systems, and adaptive learning loops to respond to evolving adversarial tactics. ^134^ From a governance standpoint, integrating AI with risk management frameworks and sector-specific compliance standards is essential for ensuring transparency, accountability, and auditability in decision-making. For instance, sectoral applications in telecommunications highlight the importance of aligning AI-based detection and mitigation tools with organizational and regulatory contexts, recognizing the interdependence of technical, human, and legal dimensions. ^135^ On the resilience axis, the framework should employ autonomous and collaborative agents capable of predictive risk assessment, cross-domain situational awareness, and integration with digital twins for pre-emptive testing of security postures in simulated environments. ^72^ These resilience mechanisms must be underpinned by zero-trust architectures and privacy-preserving learning models to safeguard sensitive datasets while enabling collaborative threat intelligence sharing. Finally, an ethical oversight layer embedding fairness, explainability, and dual-use governance ensures the trustworthiness of deployed systems, preventing misuse while preserving operational efficacy in high-stakes contexts. ^63^ This integrated analytical framework provides a structured blueprint for designing, deploying, and governing agentic AI systems in cybersecurity, bridging technical innovation with responsible stewardship. Figure 10 presents a unifying analytical framework that integrates design, governance, and resilience dimensions of agentic AI in cybersecurity. The framework maps these elements across the agentic AI lifecycle, enabling strategic alignment between architecture, oversight, and threat mitigation strategies for secure deployment.

## 6. Conclusion

This review has examined the intersection of Agentic Artificial Intelligence and cybersecurity, with a focus on cognitive autonomy, ethical governance, and quantum-resilient defense. The primary objective was to synthesize diverse literature from 2005-2025, highlighting how agentic systems characterized by autonomy, adaptability, and goal-directed reasoning are reshaping cyber defense strategies while introducing new governance and security challenges. By mapping design patterns, governance frameworks, and resilience strategies, the review identified that Agentic AI offers substantial advantages in proactive threat mitigation, continuous learning, and adaptive incident response. However, these capabilities also amplify dual-use risks, governance gaps, and the urgency for quantum-era readiness. The comparative analysis with existing frameworks, including the NIST AI RMF and EU AI Act, underscored the need for integrated governance mechanisms that align ethical principles with operational security measures. In terms of prospects, the convergence of agentic architectures with quantum-safe protocols, neurosymbolic AI, and federated learning models presents opportunities for unprecedented resilience in cybersecurity infrastructures. Nevertheless, achieving these outcomes will require coordinated policy development, technical innovation, and cross-domain knowledge transfer, drawing lessons from sectors such as aviation, finance, and healthcare. Ultimately, this review contributes to the growing discourse on aligning technological autonomy with societal values, advocating for a cybersecurity future in which Agentic AI operates as both a strategic enabler and a governed entity capable of delivering security, resilience, and trust in an increasingly complex digital ecosystem.

## Ethics and consent statement

Ethical approval and consent were not required.

## Data Availability

The supplementary materials underlying this article are openly available on Figshare 10.6084/m9.figshare.29966266.v1 ^41^: A Review of Agentic AI in Cybersecurity: Cognitive Autonomy, Ethical Governance, and Quantum-Resilient Defense: Supplementary Data. This repository contains Tables, Figures, Appendix files, Code, and Supplementary Data. All newly generated materials and supplementary datasets are available under the Creative Commons Attribution 4.0 International license (CC-BY 4.0).
