# Supplementary material for: A Review of Agentic AI in Cybersecurity: Cognitive Autonomy, Ethical Governance, and Quantum-Resilient Defense
Source: F1000Res. 2025 Sep 1;14:843. [Version 1] doi: 10.12688/f1000research.169337.1 (PMC12569510; doi:10.12688/f1000research.169337.1)
Supplement: Peer review history

# Reviewer report: Reviewer response for version 1

**DOI:** 10.5256/f1000research.186670.r422469

**Published:** 2025-10-28

**Authors:** Oluwaseun Oladeji Olaniyi

**Affiliations:** 1 University of the Cumberlands, Williamsburg, USA

This manuscript presents a detailed and forward-looking narrative review that explores how Agentic Artificial Intelligence (AAI) can transform cybersecurity through three interlinked pillars: cognitive autonomy, ethical governance, and quantum-resilient defense. It aims to synthesize developments from 2005 to 2025, bringing together academic, industry, and policy perspectives.

 The review is both timely and ambitious, tackling a complex subject that connects artificial intelligence, cyber defense, ethics, and emerging quantum technologies. It succeeds in offering a comprehensive and well-structured synthesis that not only summarizes the field but also identifies key research gaps and areas for future work.

 Summary of the Article

 The paper begins by setting the stage for why autonomy and adaptability are now essential in cybersecurity. It positions Agentic AI as a shift from traditional, reactive AI systems to proactive, self-governing, and goal-driven intelligence capable of learning and acting in dynamic cyber environments. The authors outline how static defense mechanisms are losing effectiveness against advanced persistent threats, adversarial attacks, and the coming impact of quantum computing. They explain how agentic architectures, strengthened by cognitive reasoning and neuromorphic learning, can enhance detection capabilities, improve resilience, and embed ethical oversight.

 The methodology section clearly explains the use of a narrative review approach, which is appropriate for an evolving and interdisciplinary field. The process is transparent, covering search strategies, inclusion criteria, and thematic organization. The review is structured around four research questions focusing on design patterns, governance mechanisms, resilience strategies, and implementation challenges. The discussion integrates empirical evidence, governance frameworks such as the NIST AI Risk Management Framework and the EU AI Act, and technical foundations for quantum-safe systems.

 The results and discussion present a clear picture of how Agentic AI is moving from concept to practice. The authors highlight hybrid system designs, explainable reasoning, and human–AI teaming as emerging norms, while identifying key governance models that influence accountability and compliance. The analysis of quantum-resilient defense is particularly strong, showing how post-quantum cryptography and AI-driven threat intelligence can combine to build long-term resilience. The paper concludes with an integrated framework that ties together cognitive autonomy, governance, and quantum security as the foundation for secure and ethical AI-driven cyber defense.

 Evaluation of the Review Criteria

 1. Comprehensiveness in the context of current literature: Yes

 The review demonstrates a strong grasp of multidisciplinary literature, weaving in academic, technical, and policy perspectives. The authors reference work from multiple sectors and make relevant connections to ongoing developments in standards and regulation. One area for improvement would be to include more examples of real-world applications or pilot deployments of Agentic AI systems, which would help to ground the discussion in operational practice.

 2. Accuracy and citation support: Yes

 The article is well-referenced and up to date, with sources extending to 2025. Every major claim is backed by relevant citations. The figures, tables, and analytical summaries align well with the text. A small improvement could be made by adding brief commentary on the methodological strength of key cited works, which would help to frame the robustness of the underlying evidence.

 3. Accessibility of language: Yes

 The writing is professional, clear, and easy to follow, even when dealing with complex ideas. Terms such as cognitive autonomy and ethical governance are clearly defined, and the writing balances academic precision with readability. Some sections where multiple frameworks are compared could be slightly simplified for flow and clarity.

 4. Appropriateness of conclusions: Yes

 The conclusions are logical and well supported by the findings. The authors provide a thoughtful roadmap for future research, including directions such as neurosymbolic AI and federated quantum-safe learning. It would strengthen the closing section to discuss potential implementation barriers in less developed regions or sectors where governance and funding capacity are still limited.

 Constructive Feedback and Required Revisions

 To further improve the paper and reinforce its scientific strength, I recommend the following:

- Expand the discussion on real-world or pilot deployments of Agentic AI in cybersecurity.
- Include a short critical reflection on the quality and limitations of key studies cited.
- Clarify how ethical governance frameworks can move from conceptual models to applied oversight mechanisms.
- Strengthen the section on balancing AI autonomy with human supervision, since this is critical for accountability and public trust.

Conclusion

 This is a well-structured and deeply informed review that captures both the technical and ethical dimensions of Agentic AI in cybersecurity. It brings together diverse perspectives to form a cohesive understanding of where the field stands and where it is headed. The authors show strong analytical and conceptual command, presenting insights that will benefit both researchers and practitioners. With a few enhancements focused on practical validation and methodological critique, this paper will stand as a meaningful and scientifically sound contribution to the growing literature on AI-enabled cyber defense.

 My recommendation is that we should accept with minor revisions.

Is the review written in accessible language?

Yes

Are all factual statements correct and adequately supported by citations?

Yes

Are the conclusions drawn appropriate in the context of the current research literature?

Yes

Is the topic of the review discussed comprehensively in the context of the current literature?

Yes

Reviewer Expertise:

Cybersecurity, Artificial Intelligence, Information Governance, Data Privacy, Quantum-Resilient Computing, Blockchain Technology, Cloud Security, Digital Trust, Risk Management, Cyber-Physical Systems, AI Ethics, Machine Learning, FinTech Compliance, Governance Frameworks, Cyber Resilience, Data Analytics, and Strategic Leadership in Technology Policy.

I confirm that I have read this submission and believe that I have an appropriate level of expertise to confirm that it is of an acceptable scientific standard.

# Reviewer report: Reviewer response for version 1

**DOI:** 10.5256/f1000research.186670.r422471

**Published:** 2025-10-28

**Authors:** Hassan Rehan

**Affiliations:** 1 Purdue University, West Lafayette, Indiana, USA

The manuscript, **“A Review of Agentic AI in Cybersecurity: Cognitive Autonomy, Ethical Governance, and Quantum-Resilient Defense,”** offers a well-organized narrative review of how agentic artificial intelligence (AAI) is shaping the future of cybersecurity. The authors describe Agentic AI as autonomous, goal-driven systems that can learn, adapt, and make proactive decisions in complex and changing cyber environments.

 The review is built around three main themes — **Cognitive Autonomy, Ethical Governance, and Quantum-Resilient Defense** — and does a solid job connecting technical innovation with policy and ethical considerations. It draws on literature spanning two decades (2005–2025) and touches on a wide range of areas, from reinforcement learning and neuromorphic architectures to global governance standards like the EU AI Act and ISO/IEC 42001:2023, as well as emerging quantum-safe encryption methods.

 Overall, the manuscript is clearly written and thoughtfully structured. It takes on an ambitious scope by weaving together the technical, ethical, and governance dimensions of cybersecurity into a single, cohesive framework. The authors succeed in presenting a broad yet insightful picture of how Agentic AI can be deployed securely and responsibly in practice.

Is the review written in accessible language?

Yes

Are all factual statements correct and adequately supported by citations?

Partly

Are the conclusions drawn appropriate in the context of the current research literature?

Yes

Is the topic of the review discussed comprehensively in the context of the current literature?

Yes

Reviewer Expertise:

Artificial Intelligence • Cybersecurity • Cloud Computing • Machine Learning • Ethical AI Governance • Quantum-Resilient Systems

I confirm that I have read this submission and believe that I have an appropriate level of expertise to confirm that it is of an acceptable scientific standard.
